# Supplementary material for: Complexome profiling of the Chlamydomonas psb28 mutant reveals TEF5 as an early PSII assembly factor
Source: Plant Cell. 2025 Mar 17;37(6):koaf055. doi: 10.1093/plcell/koaf055 (PMC12164590; doi:10.1093/plcell/koaf055)
Supplement: koaf055_Supplementary_Data [file koaf055_supplementary_data.zip › psb28_interactive.zip!/plots/intensity/qualityPlots/peptides/_Cre08.g378750.t1.1.html]

### Cre08.g378750.t1.1

Trivial name:   
SeqCoverage [median]: 6.40  
Mass: 71.60 kDa  
  
 

| Sequence | MissedCleavage | Mass [Da] | wtIntensity | muIntensity | Score |
| --- | --- | --- | --- | --- | --- |
| AVVADPAYAADVLR | 0 | 1429.76 | 1333.83 | 3019.26 | 74.94 |
| EALLALVAQR | 0 | 1082.64 | 278.85 | 0.00 | 63.48 |
| ELQEEVAK | 0 | 944.48 | 712.21 | 0.00 | 91.91 |
|  |  |  | 2324.9 | 3019.3 |  |
